# Evaluating student satisfaction and self-confidence across a scaffolded simulation curriculum in undergraduate nursing education

**DOI:** 10.3389/fmed.2026.1842182

**Published:** 2026-06-15

**Authors:** Anna Übelacker, Helmut Parisch, Julian Novak, Martin Ernst, Benjamin Roszipal

**Affiliations:** 1 Department of Healthcare and Nursing, Faculty of Health and Social Science, University of Applied Sciences St. Pölten, Sankt Pölten, Austria; 2 Center for Digital and Social Innovation, Department of Health Sciences, Faculty of Health and Social Sciences, University of Applied Sciences St. Pölten, Sankt Pölten, Austria; 3 Department of Health Sciences, Faculty of Health and Social Science, Institute of Health Sciences, University of Applied Sciences St. Pölten, Sankt Pölten, Austria; 4 Division of Neonatology, Pediatric Intensive Care and Neuropediatrics, Department of Pediatrics, Comprehensive Center for Pediatrics, Medical University of Vienna, Vienna, Austria

**Keywords:** curriculum evaluation, nursing education, self-confidence in learning, simulation, student satisfaction

## Abstract

**Introduction:**

Simulation in undergraduate nursing education is often evaluated as a single teaching event rather than as a coherent curriculum across multiple clinical contexts. Less is known about how students experience simulation when it is delivered as a scaffolded sequence across settings. To evaluate student satisfaction and self-confidence in learning across a scaffolded simulation curriculum delivered in four clinical settings within undergraduate nursing education.

**Methods:**

This prospective longitudinal repeated-measures study was conducted in a third-year undergraduate nursing simulation course at a university of applied sciences. The curriculum comprised four sequential blocks and combined prelearning, team-based standardized patient simulation, and PEARLS-guided debriefing. Of 135 students who completed the course in the standard format, 106 completed the Student Satisfaction and Self-Confidence in Learning Scale at least once, yielding 396 survey responses.

**Results:**

Satisfaction and self-confidence were high overall, though scores were characterized by prominent ceiling effects and negative skew (residuals skewness = −2.56). While ratings increased significantly across the sequential blocks (*p* < 0.001), sensitivity analyses excluding extreme outliers confirmed the stability of these trends.

**Conclusion:**

Students perceived the curriculum as coherent and supportive across diverse care settings. However, as curriculum blocks were delivered in a fixed order, sequence and setting effects remain confounded. Observed block differences should therefore be interpreted as reflecting cumulative familiarity rather than being uniquely attributable to the clinical settings or the scaffolded design itself.

**Study registration:**

This study was preregistered within a larger longitudinal project and later registered as a stand-alone study in the Open Science Framework (OSF) (https://doi.org/10.17605/OSF.IO/CXJ3M).

## 1 Introduction

Preparing undergraduate nursing students for clinical practice requires more than mastering isolated technical skills. Students must learn to recognize clinically relevant cues, prioritize competing demands, and make safe decisions across diverse care situations. Simulation-based education offers an important response to this challenge by allowing students to practice clinical reasoning, communication, and teamwork in a safe and structured environment without risk to patients (1–3).

In nursing education, however, simulation is often evaluated at the level of individual sessions or single-setting learning experiences, frequently using one-time post-activity surveys (3, 4). Although such evaluations provide useful information about immediate learner reactions, they offer limited insight into how students experience simulation when it is embedded within a broader curriculum and delivered across multiple clinical contexts over time (5). As a result, less is known about learner-reported outcomes in simulation curricula that are intentionally designed as longitudinal, scaffolded educational experiences rather than as isolated simulation events (6, 7).

A curriculum-level perspective is particularly relevant because nursing practice is inherently context dependent. Core processes such as assessment, prioritization, communication, and evaluation are required across settings, but the demands under which they are enacted differ substantially between, for example, acute surgical care, internal care, home or palliative care, and psychiatric or pediatric care. A scaffolded curriculum, understood as a sequenced learning design that maintains stable pedagogical supports while progressively varying context and complexity, may help students engage with this complexity (8, 9). In this context, a scaffolded curriculum refers to a sequenced learning design in which the instructional structure remains stable across blocks while the clinical context is systematically varied, enabling learners to revisit core nursing processes under progressively changing conditions. Such an approach allows learners to revisit similar reasoning processes across changing settings and may support them in distinguishing generalizable principles from setting-specific demands (7, 10). Accordingly, this study was informed by scaffolding principles and a curriculum-level view of simulation as a sequenced learning pathway rather than a series of isolated teaching events (7–10).

Based on this rationale, we implemented a simulation curriculum embedded in four sequential curriculum blocks representing core areas of undergraduate nursing education: acute surgical care, internal care, long-term/home/palliative care, and psychiatric/pediatric care. Across all blocks, the instructional sequence remained constant, combining structured prelearning with simulation-based teaching. This stable sequence was intended to prepare students for the upcoming scenarios through independent content engagement and then deepen their learning in the face-to-face setting, with particular emphasis on social and communicative aspects of nursing practice (7, 11).

Debriefing was conducted consistently using the PEARLS framework, which served both as a structured reflection method and as a central element of curricular continuity across settings (12). Accordingly, the focus of this study was not a single simulation event, but students’ learning experience across a coherent, scaffolded course curriculum.

To evaluate this curriculum-level learning experience, we focused on student satisfaction and self-confidence in learning using the Student Satisfaction and Self-Confidence in Learning Scale (SSSCL) (13–15). These outcomes were appropriate for this formative curriculum evaluation because they capture students’ subjective experience of structured, repeated simulation across varying clinical contexts and thus provide a suitable first step in evaluating whether the curriculum was experienced as supportive, coherent, and educationally meaningful.

To address these curricular challenges, the present research process was guided by the NLN Jeffries Simulation Theory, which conceptualizes simulation as a complex pedagogical system where context, design, and facilitator actions interact to influence learner outcomes. Within this framework, student satisfaction and self-confidence are recognized as primary indicators of educational effectiveness. Furthermore, the study utilized the principle of scaffolding to evaluate whether a stable instructional architecture can support students as they navigate increasing clinical complexity across diverse care settings. The aim of this study was to evaluate student satisfaction and self-confidence in learning within a scaffolded simulation curriculum delivered across four clinical settings. Specifically, we examined (1) overall levels of satisfaction and self-confidence across the course and (2) differences in these outcomes between curriculum blocks.

## 2 Methods

### 2.1 Study design and registration

**Figure 1:**
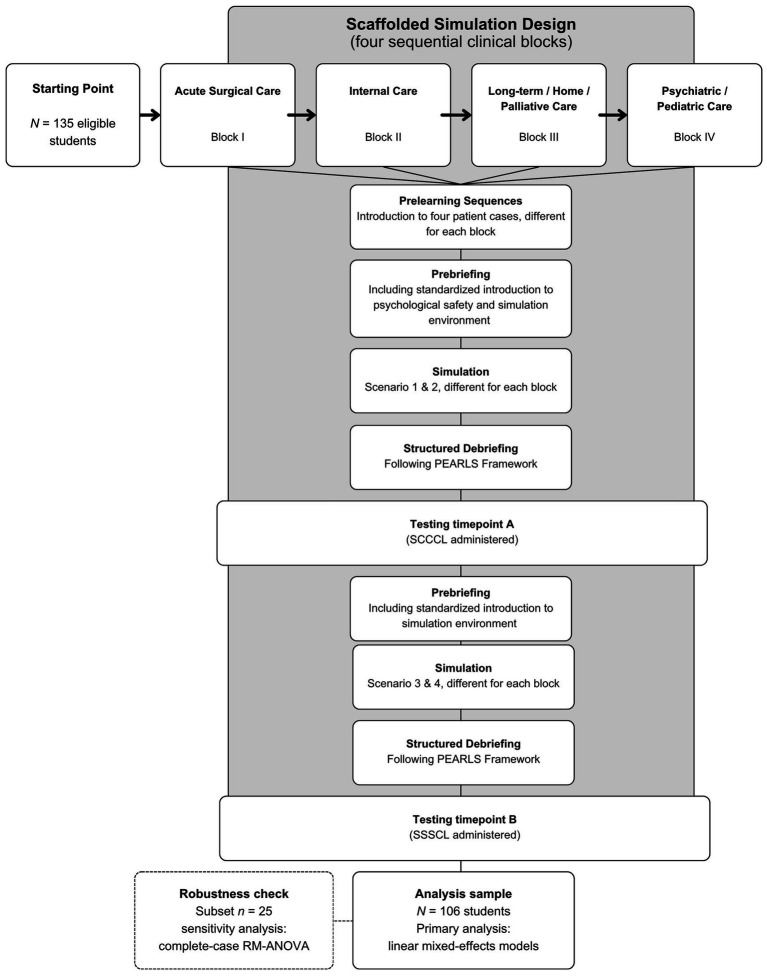
Study flowchart. The eligible study cohort comprised students who completed the course in the standard format (*N* = 135). SSSCL data were collected at two timepoints within each curriculum block. Primary analyses included all students with at least one SSSCL response (*n* = 106), whereas sensitivity analyses were restricted to complete cases across all four blocks (*n* = 25).

This study used a prospective longitudinal repeated-measures design to evaluate learner-reported outcomes across a scaffolded third-year undergraduate nursing simulation curriculum. The course comprised four sequential curriculum blocks representing different areas of nursing care: acute surgical care, internal care, long-term/home/palliative care, and psychiatric/pediatric care. Students who completed the course in the standard format participated in all four blocks. This design was considered suitable because the study aimed to examine learner-reported outcomes repeatedly across the curricular sequence and to capture changes over time within the same student cohort. The educational design was informed by scaffolding principles, with a stable instructional structure maintained across blocks while the clinical context varied (Figure 1). The evaluation was preregistered on the Open Science Framework (OSF) and included a study protocol and brief statistical analysis plan.^1^ This study is reported in accordance with the STROBE guideline for cohort studies; the completed checklist is provided in Supplementary material S1.

### 2.2 Theoretical framework

The theoretical framework guiding this study is based on the NLN Jeffries Simulation Theory and the pedagogical principle of scaffolding. The NLN Jeffries Simulation Theory conceptualizes simulation as a dynamic pedagogical system involving context, design, simulation experience, facilitator, and participants, which collectively influence learner outcomes. Within this framework, student satisfaction and self-confidence are identified as central short-term outcomes and key indicators of educational effectiveness (16, 17).

The study’s longitudinal approach was further informed by scaffolding principles, which describe a sequenced learning design that provides stable pedagogical supports while progressively varying context and complexity. In this study, “scaffolding” was operationalized by maintaining a consistent instructional architecture (Moodle-based prelearning, standardized scenarios, and PEARLS-guided debriefing) across all four clinical blocks. By keeping the methodology stable, the design aimed to reduce students’ cognitive load related to the simulation process, allowing them to focus more effectively on the increasing clinical and communicative demands of the different care settings (8).

### 2.3 Participants and setting

This study was conducted at [University name withheld for anonymity] within a third-year undergraduate nursing simulation course between October and December 2025. Recruitment occurred at the start of the course, and learner-reported outcomes were collected repeatedly across the four curriculum blocks at predefined survey timepoints. Of 144 enrolled students, 9 received recognition of prior learning and did not complete the course in its standard format. The remaining 135 students constituted the eligible study population. Inclusion criteria were enrollment in the course and written informed consent; exclusion criteria were non-completion of the course within the scheduled timeframe and/or absence of consent. Of these, 106 completed the survey at least once. Because sociodemographic data were only available as administrative cohort-level data and could not be linked to the pseudonymized survey dataset, individual-level demographic analyses and responder-nonresponder comparisons were not possible, and the number of cases reported for sociodemographic variables differ from the number of participants included.

### 2.4 Curriculum and simulation implementation

The curriculum was designed as a scaffolded simulation course delivered across four setting-specific blocks anchored in the undergraduate nursing curriculum. Across all blocks, the instructional structure remained constant while the clinical context varied. Stable instructional elements across all blocks included prelearning, prebriefing, team-based standardized patient scenarios, and structured debriefing. In line with scaffolding principles, the curriculum maintained stable pedagogical supports across blocks while systematically varying the clinical setting. Within each block, students completed four standardized patient scenarios. Before each scenario, they completed an approximately 1-h Moodle-based prelearning module per patient introducing the cases later encountered in the simulation setting and focusing on patient assessment and nursing-process reasoning. Simulation scenarios were conducted in teams of two. Scenario duration was approximately 15–20 min. Each simulation day began with a standardized prebriefing.

### 2.5 Data collection

Student satisfaction and self-confidence in learning were assessed using online surveys administered at predefined points within each simulation block. In each block, students were invited to complete the SSSCL after the first two scenarios (timepoint A) and again after the remaining two scenarios (timepoint B). Accordingly, each student could contribute up to two survey responses per block and up to eight responses across the full course. This resulted in repeated learner-reported measurements across the four curriculum blocks. Survey responses were pseudonymized using stable anonymous labels that allowed repeated responses to be linked at the individual level without collecting directly identifying information.

### 2.6 Measures

Student satisfaction and self-confidence in learning were measured using the SSSCL. The instrument comprises two subscales: satisfaction (5 items) and self-confidence in learning (8 items), rated on a 5-point Likert scale. The English version was used, with a German-language version available within the survey interface. The German translation was developed through a forward-backward translation process involving a native speaker and a faculty expert in simulation-based education.

Satisfaction and self-confidence scores were treated as continuous variables in the analyses. Subscale scores were calculated as mean scores across the respective items. Consistent with previous validation studies, item 13 was excluded from the self-confidence scale because of conceptual and psychometric concerns, resulting in a 12-item version of the instrument (16, 17). In the present sample, Cronbach’s alpha increased from 0.905 to 0.942 after exclusion of item 13.

### 2.7 Statistical analysis

Analyses followed the prespecified statistical analysis plan, which was updated prior to inferential testing to account for the hierarchical structure of the data and incomplete repeated measurements. Participant flow was summarized descriptively. Means and standard deviations were calculated overall and by curriculum block for both SSSCL subscales.

The primary outcomes were satisfaction and self-confidence in learning, operationalized as mean subscale scores. The primary explanatory variable was curriculum block, modeled as a four-level categorical variable. The primary inferential analyses examined differences in satisfaction and self-confidence across curriculum blocks. Because students could contribute multiple survey responses and survey completion varied across blocks, the data had a non-balanced repeated-measures structure with missing observations. Linear mixed-effects models were therefore used to account for within-student dependency while retaining all available data. The primary analyses included all available responses from the 106 students who completed the SSSCL at least once.

Linear mixed-effects models were estimated for satisfaction and self-confidence using restricted maximum likelihood (REML). Curriculum block (1–4) was entered as a fixed effect, and student identifier as a random intercept. Repeated measurements were modeled using an autoregressive covariance structure. Degrees of freedom were estimated using the Satterthwaite approximation. Estimated marginal means were computed for each block, and pairwise comparisons were adjusted using Bonferroni correction.

As a robustness check, complete-case repeated-measures analyses of variance (RM-ANOVA) were additionally conducted. These sensitivity analyses were restricted to participants with complete observations across all four curriculum blocks, resulting in a complete-case subset of 25 students. These sensitivity analyses showed the same directional pattern as the primary models and are reported in Supplementary material S2. All analyses were conducted using IBM SPSS Statistics, version 30. All tests were two-sided with a significance level of 0.05.

### 2.8 Missing data and exclusions

Because survey completion was voluntary, missing data were expected at both the survey and item levels. All submitted surveys were retained unless insufficient item information was available to calculate a subscale score, in which case the respective value was treated as missing. No imputation was performed. The primary mixed-model analyses therefore retained all available observations without imputing missing values. Students who completed the course but did not submit any SSSCL survey were included only in the participant flow description.

### 2.9 Ethics approval and consent to participate

The study was conducted in accordance with the Declaration of Helsinki and approved by the Ethics Committee of Lower Austria (approval number: GS3-EK-12/943–2025). All participants provided written informed consent prior to participation. Personal identifying information was stored separately from the research dataset, and all analyses were conducted on pseudonymized data.

## 3 Results

### 3.1 Participant flow and dataset

The dataset comprised 135 students who completed the course in the standard format, of whom 106 submitted the Student Satisfaction and Self-Confidence in Learning Scale (SSSCL) at least once, yielding 396 survey responses.

### 3.2 Participant characteristics

Participant characteristics are presented in Table 1. The mean age of the study cohort was 27.28 years (SD = 7.25), and 71.9% were female. These data were derived from administrative cohort-level records and describe the eligible course cohort rather than the pseudonymized survey sample.

**Table 1:** Participant characteristics.

Characteristic	Study cohort (*n* = 135)
Age, years, *M* (SD)	27.28 (7.25)
Gender, *n* (%)	
Female	97 (71.9)
Male	38 (28.1)

Age is presented as *M* (SD); categorical variables are presented as *n* (%).

### 3.3 Overall descriptive results

Overall, students rated the simulation experience very positively. Satisfaction was high (*M* = 4.52, Mdn = 4.80), and self-confidence in learning was likewise high (*M* = 4.40, Mdn = 4.50). Both variables showed marked negative skewness and evidence of ceiling effects (satisfaction: skewness = −2.73; self-confidence: skewness = −2.65), indicating consistently favorable ratings across the sample.

### 3.4 Primary analysis: Linear mixed models

**Figure 2:**
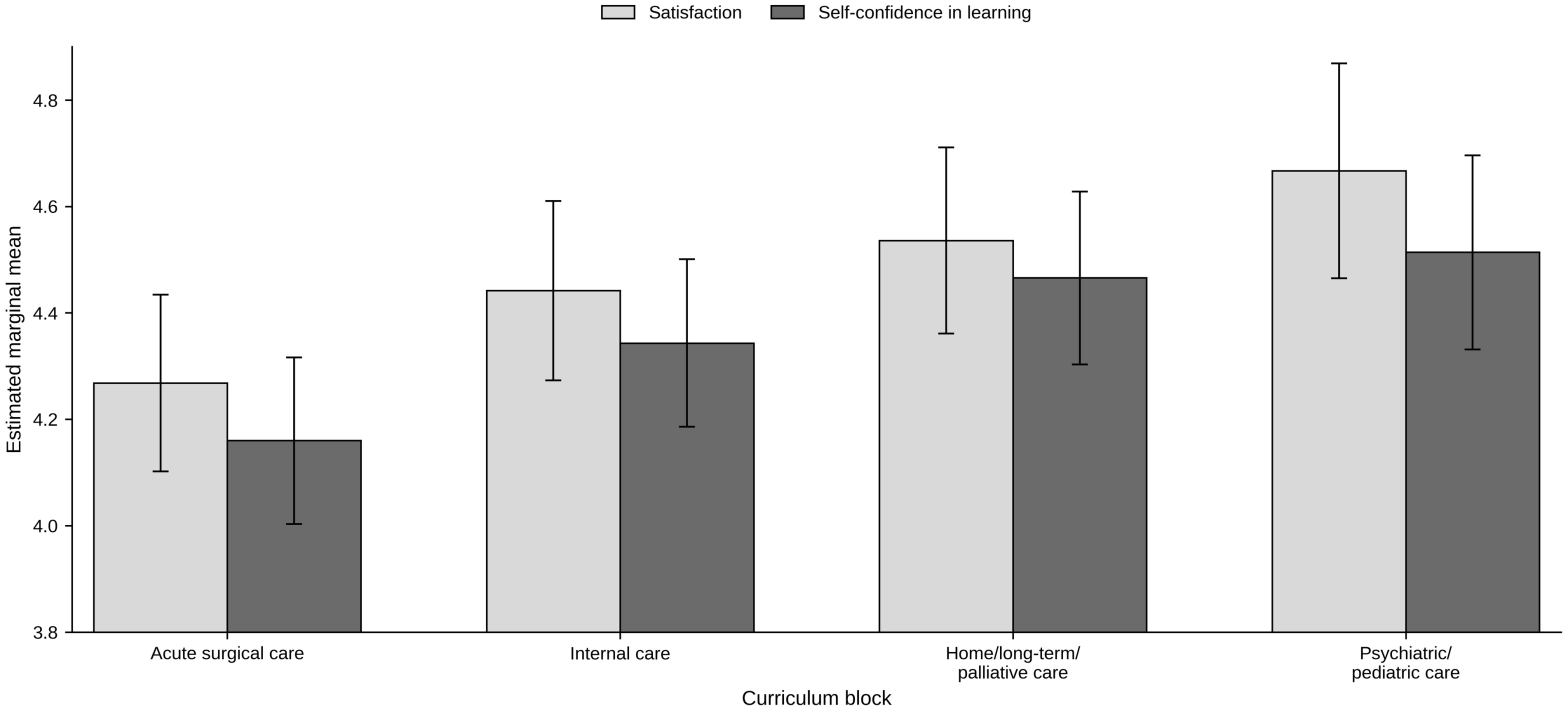
Estimated marginal means for satisfaction and self-confidence in learning across curriculum blocks. Light gray bars represent satisfaction, and dark gray bars represent self-confidence in learning. Error bars indicate 95% confidence intervals derived from the linear mixed-effects models. Higher scores indicate higher satisfaction and self-confidence.

Primary analyses used linear mixed-effects models to examine block-level differences in satisfaction and self-confidence in learning. Estimated marginal means are presented in Table 2 and Figure 2, and Bonferroni-adjusted pairwise comparisons are shown in Table 3.

**Table 2:** Estimated marginal means for satisfaction and self-confidence across curriculum blocks (*n* = 106).

Curriculum block	Mean	SE	95% CI
Acute surgical care	4.268	0.084	[4.102, 4.434]
Internal care	4.442	0.086	[4.273, 4.610]
Home/long-term/palliative care	4.536	0.089	[4.361, 4.711]
Psychiatric/pediatric care	4.667	0.103	[4.465, 4.869]
Acute surgical care	4.160	0.079	[4.003, 4.136]
Internal care	4.343	0.080	[4.186, 4.501]
Home/long-term/palliative care	4.466	0.082	[4.303, 4.628]
Psychiatric/pediatric care	4.514	0.093	[4.331, 4.696]

Values are estimated marginal means from linear mixed-effects models. CI, confidence interval. For satisfaction, pairwise comparisons showed significantly higher scores in home/long-term/palliative care than in acute surgical care (*p* = 0.007) and in psychiatric/pediatric care than in acute surgical care (*p* < 0.001). For self-confidence in learning, acute surgical care was significantly lower than internal care (*p* = 0.006), home/long-term/palliative care (*p* < 0.001), and psychiatric/pediatric care (*p* < 0.001).

**Table 3:** Bonferroni-adjusted pairwise comparisons across curriculum blocks.

Comparison	Mean difference	SE	95% CI	Adjusted *p*
Panel A. Satisfaction				
Acute surgical care vs. Internal care	−0.174	0.067	[−0.353, 0.006]	0.064
Acute surgical care vs. Home/long-term/palliative care	−0.268	0.082	[−0.486, −0.050]	0.007
Acute surgical care vs. Psychiatric/pediatric care	−0.399	0.100	[−0.669, −0.129]	< 0.001
Internal care vs. Home/long-term/palliative care	−0.095	0.072	[−0.287, 0.098]	1.000
Internal care vs. Psychiatric/pediatric care	−0.225	0.097	[−0.483, 0.033]	0.125
Home/long-term/palliative care vs. Psychiatric/pediatric care	−0.131	0.090	[−0.370, 0.109]	0.881
Panel B. Self-confidence in learning				
Acute surgical care vs. Internal care	−0.184	0.055	[−0.330, −0.038]	0.006
Acute surgical care vs. Home/long-term/palliative care	−0.306	0.067	[−0.485, −0.127]	< 0.001
Acute surgical care vs. Psychiatric/pediatric care	−0.354	0.083	[−0.578, −0.130]	< 0.001
Internal care vs. Home/long-term/palliative care	−0.122	0.058	[−0.278, 0.034]	0.229
Internal care vs. Psychiatric/pediatric care	−0.170	0.079	[−0.383, 0.042]	0.202
Home/long-term/palliative care vs. Psychiatric/pediatric care	−0.048	0.073	[−0.244, 0.148]	1.000

Values are Bonferroni-adjusted pairwise comparisons derived from the linear mixed-effects models. CI, confidence interval; SE, standard error. Mean differences and 95% confidence intervals refer to the contrast between the first and second curriculum block listed in each comparison. Negative values indicate lower estimated scores in the first block than in the second block.

Residual diagnostics for the linear mixed-effects models were conducted by visual inspection of histograms and Q-Q plots of the residuals. The residuals exhibited a significant deviation from normality (Shapiro–Wilk *p* < 0.001) and a marked negative skewness (skewness = −2.56), consistent with the prominent ceiling effects observed in student ratings. To assess the impact of extreme values, a sensitivity analysis was performed by excluding observations with standardized residuals exceeding 3 standard deviations (*n* = 8 observations removed). The results of this filtered model were consistent with the primary analysis, with curriculum block maintaining a significant effect on learner outcomes (*p* < 0.001), thereby confirming the robustness of the findings against individual outliers.

### 3.5 Student Satisfaction

For satisfaction, the model showed a significant effect of block, *F*(3, 128.71) = 6.05, *p* < 0.001. Estimated marginal means increased across blocks, from acute surgical care (*M* = 4.27, SE = 0.08) to internal care (*M* = 4.44, SE = 0.09), home/long-term/palliative care (*M* = 4.54, SE = 0.09), and psychiatric/pediatric care (*M* = 4.67, SE = 0.10). Bonferroni-adjusted pairwise comparisons indicated significantly higher satisfaction in home/long-term/palliative care than in acute surgical care (*p* = 0.007) and in psychiatric/pediatric care than in acute surgical care (*p* < 0.001; Tables 2 and 3, Figure 2). No other pairwise differences were significant.

### 3.6 Self-Confidence in Learning

A comparable pattern was observed for self-confidence in learning. The mixed model indicated a significant effect of block, *F*(3, 122.23) = 8.66, *p* < 0.001. Estimated marginal means increased across blocks, with the lowest values in acute surgical care (*M* = 4.16, SE = 0.08), followed by internal care (*M* = 4.34, SE = 0.08), home/long-term/palliative care (*M* = 4.47, SE = 0.08), and psychiatric/pediatric care (*M* = 4.51, SE = 0.09). Bonferroni-corrected pairwise comparisons indicated that self-confidence was significantly lower in acute surgical care than in internal care (*p* = 0.006), home/long-term/palliative care (*p* < 0.001), and psychiatric/pediatric care (*p* < 0.001; Tables 2 and 3, Figure 2). The remaining pairwise differences were not significant.

As a robustness check, repeated-measures ANOVAs based on complete cases only were additionally conducted. Because this approach excluded participants with missing values at any measurement point, the effective sample size was substantially reduced compared with the mixed-model analysis. The repeated-measures analyses showed the same directional pattern of increasing ratings across blocks, although effects were smaller and only marginally significant. Detailed complete-case RM-ANOVA results are reported in Supplementary material S2.

## 4 Discussion

This study examined student satisfaction and self-confidence in learning across a scaffolded simulation curriculum delivered in four clinical settings within undergraduate nursing education. Rather than evaluating simulation as an isolated teaching event, the study adopted a curriculum-level perspective by assessing learner-reported outcomes repeatedly across four sequential blocks while maintaining a stable instructional structure. Overall, students reported high levels of satisfaction and self-confidence throughout the course. Across blocks, both outcomes increased gradually, with the highest values observed in the final block. Significant block effects were identified for both satisfaction and self-confidence. Pairwise comparisons showed significantly higher satisfaction in home/long-term/palliative care and psychiatric/pediatric care than in acute surgical care, whereas self-confidence in acute surgical care was significantly lower than in all later blocks. Taken together, these findings suggest that the curriculum was experienced as a supportive and coherent learning environment across varying clinical contexts. This interpretation is consistent with recent evidence suggesting that simulation-based learning in nursing education is commonly associated with high learner satisfaction, greater confidence, and positive perceptions of educational value when embedded in well-designed learning structures (1, 2, 4).

A key contribution of this study lies in its curricular perspective. Recent nursing simulation research still frequently evaluates discrete simulation events, short-term post-activity outcomes, or single-course interventions rather than sustained learner experience across a structured curriculum (4). By contrast, the present study focused on how students experienced a repeated simulation structure embedded across multiple curriculum blocks. Emerging work on scaffolded and longitudinal simulation likewise suggests that repeated, sequenced simulation may function not only as a teaching method, but also as a curriculum design strategy that can support progressive integration of knowledge and practice over time (9, 10). In this sense, the present findings are particularly relevant for competency-oriented curriculum design and formative evaluation (18), because they illuminate how learners perceive continuity and support across a sequenced simulation program rather than within isolated teaching events (18, 19).

The consistently high satisfaction ratings across all blocks suggest that students responded positively to the overall instructional design. Satisfaction remained slightly higher than self-confidence across the curriculum, which is educationally plausible. Whereas satisfaction reflects learners’ appraisal of the educational experience, self-confidence may be more sensitive to uncertainty, challenge, and the demands of applying knowledge under pressure. The observed combination of high satisfaction and somewhat lower, though still high, self-confidence may therefore represent a realistic pattern: students perceived the learning environment as supportive while still recognizing the complexity of clinical decision-making across settings.

The gradual increase in both satisfaction and self-confidence across the four curriculum blocks is particularly relevant for interpreting the curriculum design. From a scaffolding perspective, this pattern may indicate that the stable instructional sequence increased students’ familiarity with the learning architecture, allowing them to focus more fully on the clinical and communicative demands of the scenarios. This interpretation is especially plausible for self-confidence, which was lowest in acute surgical care and significantly higher in all subsequent blocks. At the same time, these findings should not be interpreted as evidence that the curriculum itself caused improved competence or confidence, but rather as indicating a more favorable learner-reported experience in later blocks. One possible explanation is that repeated exposure to the same instructional logic may have supported growing confidence in applying the nursing process across changing contexts. Recent repeated-measures and longitudinal simulation studies support this interpretation by suggesting that sequenced simulation may be associated with progressive gains in confidence, clinical judgment, and perceived readiness for practice (7, 10, 11).

At the same time, the observed block differences should be interpreted cautiously. The higher ratings in later blocks cannot be attributed unequivocally to the characteristics of those settings themselves. An alternative explanation is that the observed pattern reflects a sequence effect: because these blocks occurred later in the curriculum, students may have entered them with greater familiarity, reduced uncertainty, and more experience with the overall course structure. The present design therefore does not allow clear separation of setting-specific effects from order effects. This distinction is central to the interpretation of the findings: the higher ratings observed in psychiatric/pediatric care, for example, may reflect the specific educational characteristics of that block, the cumulative effects of progression through the curriculum, or a combination of both. This distinction is important for interpretation and for future curriculum research, because recent scaffolded simulation studies have likewise highlighted the importance of sequencing and longitudinal integration when interpreting learner outcomes across repeated experiences (9, 10).

The findings also support the pedagogical value of aligning learning modalities across the curriculum. In this course, desk-based prelearning introduced students to the patient cases in advance and supported preparation in professional knowledge and methodological skills. The face-to-face simulation sessions then shifted the focus toward enactment, teamwork, and communication under contextual constraints, while PEARLS-guided debriefing provided a stable reflective structure across all blocks. This alignment may partly account for why learner-reported outcomes remained high despite variation in context. For educators, the findings highlight the value of maintaining a stable instructional structure across simulation blocks, as this may support learner orientation and allow greater attention to setting-specific clinical and communicative demands. In practical terms, this may involve using the same prelearning format, a consistent prebriefing structure, and a common debriefing approach or script across blocks so that students can orient themselves to the learning process while adapting to changing clinical content. These findings support the value of integrated curricular designs that combine prelearning and simulation rather than treating simulation as a standalone event (20). Recent evidence suggests that prebriefing and other presimulation preparation activities may positively influence learner readiness, engagement, and learning outcomes (21, 22). Debriefing likewise remains central to reflection, meaning-making, and professional development in simulation-based education, and structured approaches such as PEARLS can provide consistency across repeated simulation experiences (23, 24). Even though the present study does not demonstrate competence development or transfer directly, it suggests that students experienced the transition from preparation to enactment to reflection as coherent and educationally meaningful. The repeated use of standardized patients may also have contributed to the positive learner experience by supporting authentic, communication-focused learning across blocks (25, 26).

Several limitations should be considered. First, the study relied on learner-reported outcomes and therefore assessed perceived satisfaction and self-confidence rather than objective competence, performance, or transfer to clinical practice. These constructs are useful indicators of curriculum acceptability and perceived learning support, but they should not be interpreted as direct indicators of clinical capability (1, 27). Second, although 106 of 135 eligible students who completed the mandatory curriculum submitted at least one survey, only 25 students provided complete survey data across all four blocks. This high rate of attrition regarding the voluntary evaluation and the resulting non-response bias represents a significant threat to the findings, as it is unknown whether the characteristics or motivations of these persistent responders differed from those who did not complete all surveys. Furthermore, because sociodemographic data could not be linked to individual pseudonymized responses, a formal comparison between responders and non-responders was not possible. Third, although facilitators were trained and the PEARLS framework was consistently employed to provide curricular continuity, the study did not formally monitor debriefing fidelity using validated tools. This is a significant limitation, as the NLN Jeffries Simulation Theory identifies the facilitator’s actions during debriefing as a central determinant of participant outcomes. It is possible that the observed increases in later blocks were partially driven by a ‘facilitator calibration effect’: as the semester progressed, educators may have become more attuned to the specific needs of this cohort. Furthermore, as students became more familiar with the PEARLS reflective phases, the ‘pedagogical dose’ of each session might have effectively increased. Without formal fidelity assessments, we cannot rule out that differences in facilitation quality contributed to the favorable ratings in later settings, independent of the scaffolded curriculum design itself (6, 24, 28). This limitation is particularly relevant because variation in debriefing quality across blocks may have contributed to the observed block differences and therefore cannot be ruled out as an alternative explanation alongside sequence or setting effects. Fourth, the statistical assumptions of the linear mixed-effects models were partially constrained by the data distribution. Specifically, the marked negative skewness and prominent ceiling effects resulted in non-normally distributed residuals. While linear mixed models are generally robust to violations of normality, the strong clustering of scores at the upper end of the scale suggests that the SSSCL instrument may have limited sensitivity to differentiate further improvements in this high-performing cohort. Nevertheless, sensitivity analyses excluding extreme outliers demonstrated that the observed trends were stable, supporting the reliability of the reported block differences within the constraints of the fixed curriculum sequence.

These limitations also inform the outlook for future research. Most importantly, future studies should examine whether differences across curriculum blocks are primarily attributable to the specific care settings or to the sequence in which those settings are encountered. This could be addressed through alternative sequencing designs, counterbalanced block structures, or across-cohort comparisons. In addition, curriculum-level evaluation should be linked to objective outcomes such as structured performance assessments, clinical judgment measures, and indicators of longitudinal skill retention. Qualitative data could further enrich understanding of why learners feel confident in particular blocks and which design elements most strongly support their learning. Future research should also examine how specific instructional components – such as prelearning design, prebriefing consistency, and debriefing fidelity – shape learner experience within scaffolded simulation curricula. Recent scholarship on scaffolded simulation, prebriefing standards, and curriculum integration suggests that this kind of programmatic evaluation is increasingly necessary if simulation is to be understood as a curricular pathway rather than a series of isolated educational events (9, 10, 22).

## 5 Conclusion

This study provides a curriculum-level evaluation of a scaffolded simulation course delivered across four clinical settings in undergraduate nursing education. Students reported consistently high satisfaction and self-confidence, and both outcomes differed significantly across curriculum blocks, with more favorable ratings in later blocks than in acute surgical care. These findings suggest that a stable instructional structure integrating prelearning, simulation, and structured debriefing may support a coherent and positively experienced learning pathway across varying care contexts. From an educational perspective, the results highlight the value of evaluating simulation as a sequenced curricular design rather than as a series of isolated teaching events. Future research should clarify whether the observed differences primarily reflect setting-specific characteristics, sequence effects, or both, and should relate learner-reported outcomes to objective measures of performance and longitudinal development.

## Data Availability

The original contributions presented in the study are included in the article/Supplementary material, further inquiries can be directed to the corresponding author.

## Footnotes

1
https://doi.org/10.17605/OSF.IO/CXJ3M

## Supplementary material

SUPPLEMENTARY MATERIAL S1 STROBE statement checklist.
