# Supplementary material for: Evaluating student satisfaction and self-confidence across a scaffolded simulation curriculum in undergraduate nursing education
Source: Front Med (Lausanne). 2026 Jun 15;13:1842182. doi: 10.3389/fmed.2026.1842182 (PMC13310688; doi:10.3389/fmed.2026.1842182)
Supplement: Supplementary file 2 [file Supplementary_file_2.docx]

Supplementary Material S2

**Sensitivity analysis using repeated-measures ANOVA**

As a sensitivity analysis, complete-case repeated-measures ANOVAs were conducted to examine changes across the four curriculum blocks (see Table S2 and Figure S1). Because these analyses required complete data across all measurement occasions, listwise deletion reduced the analytic sample to 25 students. For satisfaction, Mauchly’s test indicated that the assumption of sphericity was violated, *W* = .357, χ²(5) = 23.41, *p* < .001. Therefore, Greenhouse–Geisser-corrected results are reported. The analysis showed a significant effect of block on satisfaction, *F*(2.10, 50.40) = 3.14, *p* = .049, partial η² = .116, indicating a medium effect. Within-subject contrasts further showed a significant linear trend across blocks, *F*(1, 24) = 5.37, *p* = .029, partial η² = .183, consistent with increasing satisfaction scores from Block 1 (*M* = 4.42) to Block 4 (*M* = 4.81). Corresponding estimated means for satisfaction and self-confidence are presented in Table S2 and Figure S1. Because repeated-measures ANOVA excludes all cases with missing data at any measurement occasion, linear mixed-effects models were additionally estimated in the main analysis to retain all available observations and provide a more robust approach to incomplete repeated-measures data.

**Table S1:** Estimated Means and 95% Confidence Intervals Across Curriculum Blocks From Complete-Case Repeated-Measures ANOVAs (*n* = 25)

| **Panel A. Satisfaction** | | | | |
| --- | --- | --- | --- | --- |
| **Curriculum Block** | **Mean** | **SE** | **95% CI Lower** | **95% CI Upper** |
| Acute surgical care | 4.420 | 0.188 | 4.033 | 4.807 |
| Internal care | 4.604 | 0.168 | 4.257 | 4.951 |
| Home/long-term/palliative care | 4.700 | 0.136 | 4.419 | 4.981 |
| Psychiatric/pediatric care | 4.808 | 0.068 | 4.668 | 4.948 |
| **Panel B. Self-confidence in learning** | | | | |
| **Curriculum Block** | **Mean** | **SE** | **95% CI Lower** | **95% CI Upper** |
| Acute surgical care | 4.270 | 0.172 | 3.915 | 4.625 |
| Internal care | 4.540 | 0.144 | 4.243 | 4.837 |
| Home/long-term/palliative care | 4.593 | 0.134 | 4.315 | 4.870 |
| Psychiatric/pediatric care | 4.580 | 0.092 | 4.390 | 4.770 |
| Acute surgical care | 4.270 | 0.172 | 3.915 | 4.625 |

**Note:** Values are estimated means from complete-case repeated-measures ANOVAs. CI = confidence interval. Estimates are based on participants with complete observations across all curriculum blocks.

**Figure S1:** Estimated means for satisfaction and self-confidence in learning across curriculum blocks based on complete-case repeated-measures ANOVAs.


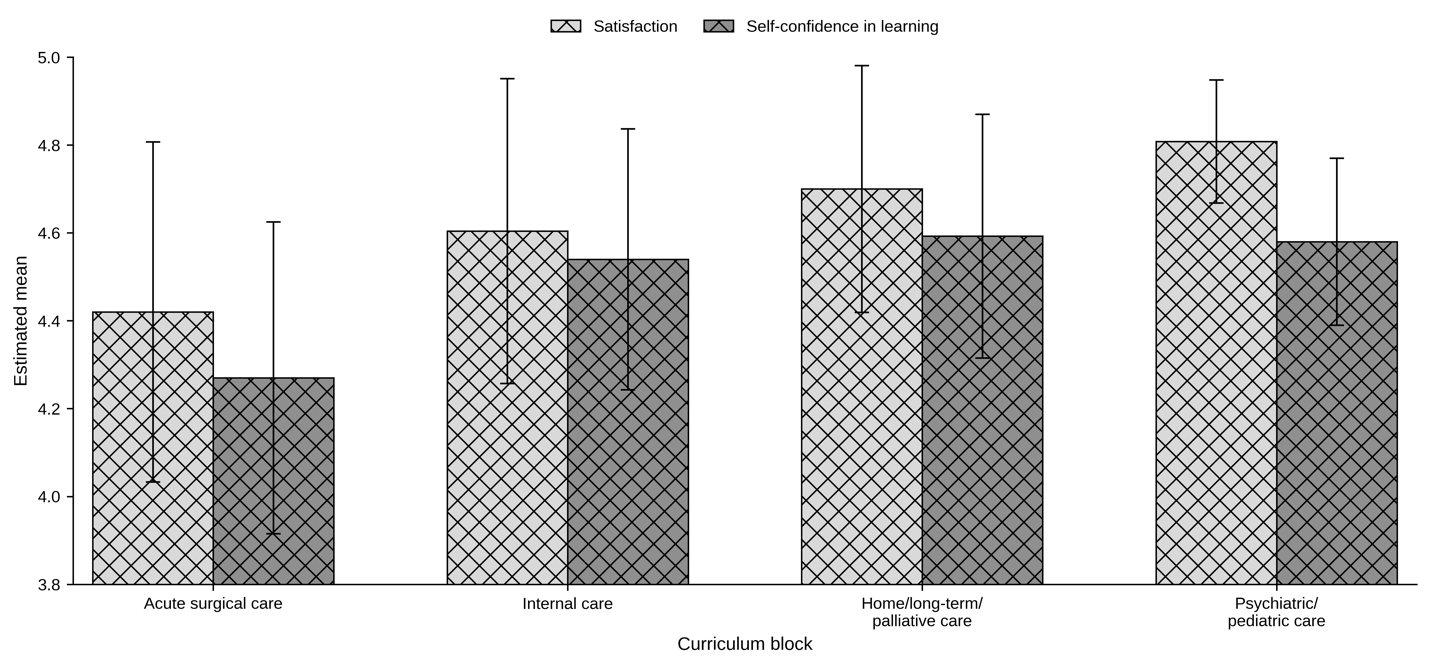


**Note:** Bars represent estimated means from complete-case repeated-measures ANOVAs. Light gray bars indicate satisfaction, and dark gray bars indicate self-confidence in learning. Error bars represent 95% confidence intervals. Estimates are based on participants with complete observations across all curriculum blocks (*n* = 25).
